# Influenza-like illness in an urban community of Salvador, Brazil: incidence, seasonality and risk factors

**DOI:** 10.1186/s12879-016-1456-8

**Published:** 2016-03-15

**Authors:** Carlos R. Oliveira, Gisela S. R. Costa, Igor A. D. Paploski, Mariana Kikuti, Amelia M. Kasper, Monaise M. O. Silva, Aline S. Tavares, Jaqueline S. Cruz, Tássia L. Queiroz, Helena C. A. V. Lima, Juan Calcagno, Mitermayer G. Reis, Daniel M. Weinberger, Eugene D. Shapiro, Albert I. Ko, Guilherme S. Ribeiro

**Affiliations:** 1 Department of Pediatrics, School of Medicine, Yale University, New Haven, USA; 2 Centro de Pesquisas Gonçalo Moniz, Fundação Oswaldo Cruz, Ministério da Saúde, Rua Waldemar Falcão, 121, Candeal, 40296-710 Salvador, Brazil; 3 Instituto de Saúde Coletiva, Universidade Federal da Bahia, Salvador, Brazil; 4 Department of Epidemiology of Microbial Diseases, School of Public Health, Yale University, New Haven, USA; 5 Faculdade de Medicina, Universidade Federal da Bahia, Salvador, Brazil; 6 Department of Investigative Medicine, School of Medicine, Yale University, New Haven, USA

**Keywords:** Influenza-like Illness, Tropics, Brazil, Incidence and Seasonality

## Abstract

**Background:**

Our understanding of the epidemiology of influenza is limited in tropical regions, which in turn has hampered identifying optimal region-specific policy to diminish disease burden. Influenza-like illness (ILI) is a clinical diagnosis that can be used as a surrogate for influenza. This study aimed to define the incidence and seasonality of ILI and to assess its association with climatic variables and school calendar in an urban community in the tropical region of Salvador, Brazil.

**Methods:**

Between 2009 and 2013, we conducted enhanced community-based surveillance for acute febrile illnesses (AFI) among patients ≥5 years of age in a slum community emergency unit in Salvador, Brazil. ILI was defined as a measured temperature of ≥37.8 °C or reported fever in a patient with cough or sore throat for ≤7 days, and negative test results for dengue and leptospirosis. Seasonality was analyzed with a harmonic regression model. Negative binomial regression models were used to correlate ILI incidence with rainfall, temperature, relative humidity and the number of days per month that schools were in session while controlling for seasonality.

**Results:**

There were 2,651 (45.6 % of 5,817 AFI patients) ILI cases with a mean annual incidence of 60 cases/1,000 population (95 % CI 58–62). Risk of ILI was highest among 5–9 year olds with an annual incidence of 105 cases/1,000 population in 2009. ILI had a clear seasonal pattern with peaks between the 35–40^th^ week of the year. ILI peaks were higher and earlier in 5–9 year olds compared with >19 year olds. No association was seen between ILI and precipitation, relative humidity or temperature. There was a significant association between the incidence of ILI in children 5–9 years of age and number of scheduled school days per month.

**Conclusions:**

We identified a significant burden of ILI with distinct seasonality in the Brazilian tropics and highest rates among young school-age children. Seasonal peaks of ILI in children 5–9 years of age were positively associated with the number of school days, indicating that children may play a role in the timing of seasonal influenza transmission.

**Electronic supplementary material:**

The online version of this article (doi:10.1186/s12879-016-1456-8) contains supplementary material, which is available to authorized users.

## Background

Influenza is a vaccine preventable disease, yet every year, it is estimated to cause over 500,000 deaths worldwide [1]. Influenza disproportionally affects individuals from resource-poor settings with children and the elderly at highest risk [2]. Among all pediatric deaths from influenza, 99 % occur in developing countries [3]. Assessing the true incidence of influenza in low and middle-income countries is a challenge due to the lack of access to diagnostic testing. Influenza-like illness (ILI) is a clinical diagnosis that can be used as a surrogate for influenza in epidemiological studies. The Centers for Disease Control and Prevention (CDC) recommend using the case definition for ILI (fever plus cough or sore throat) for surveillance of influenza [4]. Multiple variations of these definitions have been developed and are used in clinical research. Including reported or measured fever in the case definition of ILI substantially increases the sensitivity of identifying influenza in developing countries. Although the sensitivity and specificity of ILI case definition varies considerably year to year, estimates from developing countries suggest that the use of an ILI case definition that includes “reported or measured fever” has a sensitivity ranging between 67–87 % and a specificity range of 53–70 % for identifying influenza [5–7].

The epidemiology of influenza has been well defined in temperate climates, with predictable peaks during the winter months. In contrast, little is known about either the burden of disease or the seasonality of influenza in the tropics. Of the limited data that are available, findings have been inconsistent, with some studies identifying a single annual peak, others finding two annual peaks and still others finding no distinct seasonality [8–11].

Several studies have investigated associations between rates of influenza and climactic variables such as rainfall, temperature and relative humidity [12–16]. However, there are insufficient data demonstrating the impact of these environmental factors on the seasonality of influenza in the tropics where temperature and humidity are higher. Additionally, some studies have found that winter school breaks were significantly associated with a reduction in the incidence of ILI among school-age children, though all of these observations were in temperate regions [17].

The burden and seasonality of influenza in Brazil is of particular interest given that the country’s climate differs greatly from the northern tropical regions to more temperate subtropical regions in the south. In Brazil, the influenza vaccines have been offered to the elderly population (>65 years of age) since 1999. The target age group was expanded to include the population >60 years of age in 2000, and later further expanded to include children from 6 months to 5 years of age, healthcare workers, pregnant and puerperal women and other high-risk groups (prisoners, indigenous, and those with certain chronic diseases). The vaccine is given in annual mass campaigns with an estimated coverage of over 80 % [18]. Though these campaigns are typically held in April and May, few studies have been published on the incidence and seasonality of influenza in Brazil. Of the published reports, the findings have been inconsistent, with significant differences in both timing and magnitude of the epidemic peaks [9, 13, 19]. Information on disease burden and seasonality is important for determining both the need for and the optimal timing of administration of the influenza vaccine.

The objectives of this study were to define the incidence and seasonality of ILI in a tropical urban community in Salvador, Brazil. In addition, we aimed to identify potential contributors to the seasonality of ILI, such as meteorological factors and the number of days local schools were in session.

## Methods

### Study site and population

The study was conducted in Pau da Lima, an urban slum community located in Salvador, a city in tropical, Northeastern Brazil with a population of 2.6 million people in 2010 (IBGE census) [20]. The data used in this study were originally collected in this community by the Oswaldo Cruz Foundation (Fiocruz) as part of an enhanced surveillance study for acute febrile illness (AFI), whose aim was to better understand the epidemiology of leptospirosis and dengue in slum communities. Surveillance was based at the São Marcos Emergency Center (SMEC), which is the sole public urgent care facility within Pau da Lima.

To estimate the incidence and seasonality of ILI, two distinct population bases were used (one for incidence calculations and the other for seasonality estimates). The first population base was the entire Pau da Lima community, which includes 98 census tracts and 76,352 inhabitants according to the National Census in 2010 [20, 21]. Seasonality analyses were done using this population base. The second population base was the Fiocruz cohort site, an area with 12,908 inhabitants nested within the Pau da Lima community [22, 23]. The Fiocruz cohort site has a population with well-defined demographics that was established by a collaborative Yale-Fiocruz research group to conduct prospective cohort studies. SMEC is adjacent to the Fiocruz cohort site; 84 % of the Fiocruz cohort residents report that in the event of a febrile illness requiring medical care, they would go to SMEC. Incidences calculations were done using the Fiocruz cohort population base. The Institutional Review Boards at Yale University, the Oswaldo Cruz Foundation, and the Brazilian National Committee for Ethics in Research approved the project.

### Surveillance

From April 1, 2009 to March 31, 2013, acute febrile illness surveillance was conducted at the SMEC, Monday to Friday, from 07:30 AM to 04:00 PM. During this period, patients were identified upon arrival and invited to participate if they were: 1) five years of age or older; 2) residents of Pau da Lima; and 3) reported fever or had a measured temperature ≥37.8° in the previous 21 days. Written informed consent was obtained from the patient or parent. Using a standardized questionnaire, data on self-reported symptoms and demographics and socio-economic characteristics were collected through interviews. An acute-phase blood sample was collected at the time of enrollment and a convalescent-phase sample was obtained after 14 days. If the patient did not return to the SMEC for the convalescent-phase sample evaluation, a team visited the patient at home and collected the sample. Additionally, medical records from all the patients who presented to the SMEC with fever were reviewed to identify those who met the inclusion criteria for the study, but were not enrolled because they sought treatment outside of the study hours or refused to participate.

### Laboratory methods and case definitions

An ILI case was defined based on a modified version of the CDC criteria for influenza surveillance in developing countries and included measured temperature of ≥37.8 °C or reported fever with cough or sore throat for ≤7 days from symptom onset. Patients were not considered to have ILI if they were diagnosed with probable or confirmed dengue or leptospirosis.

Blood samples were stored at −20 °C for serological testing and at −70 °C for reverse transcriptase-polymerase chain reaction (RT-PCR) assays. Both acute and convalescent phase sera were tested for leptospirosis and dengue. Confirmed leptospirosis was defined as a four-fold or greater rise or seroconversion (from negative to ≥1:200) in microscopic agglutination test (MAT) titer between paired samples, or a titer of ≥1:800 in a single sample. Probable leptospirosis was defined as low titer (1:100–1:400) agglutination in either the acute or convalescent sample. Confirmed dengue was defined as seroconversion by enzyme-linked immunoabsorbent assay (ELISA) *(Panbio Diagnostics, Brisbane, Australia*) of immunoglobulin M (IgM), positive RT-PCR [24] assay or a positive non-structural protein 1 (NS1) test by ELISA *(Panbio Diagnostics, Brisbanen Australia*). Probable dengue was defined as a positive IgM-ELISA in either the acute or convalescent sample.

### Statistical analysis

Study years started on April 1^st^ and ended on March 31^st^ of each year. Differences between enrolled and not enrolled patients were evaluated with ***χ***^2^ tests for categorical variables and with Mann Whitney *U* test for continuous variables. Statistical significance was defined as a *P*-value < 0.05 (two-tailed). Incidence rates with 95 % confidence intervals were calculated as previously described by Merril et al. [25]. Given that our surveillance did not occur 24 h a day or 7 days a week, a formula was applied to the measured cases of ILI in order to adjust for the days and hours not sampled. To do this, we adjusted the actual measured incidence in the sample for the proportion of all potential AFI patients seen at the SMEC every week, month, or year with the following formula:

$$ \mathbf{Adjusted}\ \mathbf{Age}-\mathbf{Specific}\ \mathbf{I}\mathbf{ncidence} = {\mathbf{I}}_{\mathbf{measured}}/\ \left(\mathbf{Enrolle}{\mathbf{d}}_{\mathbf{AF}}/\mathbf{Tota}{\mathbf{l}}_{\mathbf{AF}\mathbf{I}}\right) $$

where I_measured_ is the measured incidence in the sample (within a specific age-strata: 5–9, 10–19, 20–29, 30–39, 40–49 and >49 year olds), Total_AFI_ is that total number of cases of AFI in the same age strata that came to the SMEC and Enrolled_AFI_ is the number of AFI cases enrolled in the same age strata.

Incidences were calculated using the Fiocruz cohort population base using data from the Fiocruz census. The residence of all enrolled patients was geocoded into a database and patients who resided within the Fiocruz cohort site were identified from this database. Incidence estimates with 95 % confidence intervals (95 % CI) were calculated from the estimated number of cases by total population and stratified by age group. Data were analyzed using Epi-Info (version 3.5.3, Centers for Disease Control and Prevention, Atlanta, GA) and Stata statistical software 12.0 (StataCorp, College Station, TX).

### Time series analysis

The population base composed of residents from the entire Pau da Lima area (which includes the nested Fiocruz cohort area) was used to assess seasonality of ILI. Seasonality was analyzed with a harmonic regression model [26]. We fit the weekly or monthly ILI time series using a Negative Binomial regression model (log-linked) including sine and cosine (harmonic) terms. The main advantages of this method are its flexibility of terms, with the ability to include annual and biannual peaks, and its ability to control for confounders. Given that some of the ILI cases will likely be false-negative for dengue, we included the known dengue time series as a covariate in the models, allowing us to estimate the fraction of ILI cases that are unreported cases of dengue and subtract them from the signal. We built both monthly and weekly models with variable peaks (annual, biannual, or both), with and without dengue terms. We used the Akaike's Information Criterion (AIC) to select the best model. AIC estimates from the alternative models are presented in Additional file 1: Table S1. The final model had both annual and biannual sine and cosine variables as well as the dengue variable in both monthly and weekly intervals as follows:

$$ \mathbf{Incidence} = \mathbf{exp}\left\{{\boldsymbol{\upbeta}}_{\mathbf{0}} + {\boldsymbol{\upbeta}}_{\mathbf{1}}\_\mathbf{cos}\left(\mathbf{\O}\right) + {\boldsymbol{\upbeta}}_{\mathbf{2}}\_\mathbf{sin}\left(\mathbf{\O}\right) + {\boldsymbol{\upbeta}}_{\mathbf{3}}\_\mathbf{cos}\left(\boldsymbol{\Phi} \right)+{\boldsymbol{\upbeta}}_{\mathbf{4}}\_\mathbf{sin}\left(\boldsymbol{\Phi} \right) + {\boldsymbol{\upbeta}}_{\mathbf{5}\_}\mathbf{dengue} + \boldsymbol{\upvarepsilon} \right\} $$

Where β_0_ represents the intercept or baseline level of ILI, β_1_ – β_4_ are the coefficients of the harmonic, Ø =2π*week/52.25 Φ = 2π*week/26.1 [representing annual (every 52.25 weeks) and biannual (every 26.1 weeks) epidemics], and β_5_ represents incidence of dengue.

Predicted weekly or monthly cases were calculated from the best model. In addition, average peak timing and intensity were estimated from the predicted curve. The average peak timing (in weeks) can be estimated from the harmonic terms as follows:

$$ \mathbf{Average}\ \mathbf{peak}\ \mathbf{timing} = \mathbf{52.25}\ \left\{\mathbf{1} - \left( - \mathbf{arctan}\ \left({\boldsymbol{\upbeta}}_{\mathbf{1}/}{\boldsymbol{\upbeta}}_{\mathbf{2}}\right)/\boldsymbol{\uppi} \right)/\mathbf{2}\right\} $$

The peak intensity (the mean difference between peak and nadir of the harmonic curves) can be estimated with the following [26]:

$$ \mathbf{Average}\ \mathbf{peak}\ \mathbf{intensity} = \mathbf{exp}\ \left\{\ {\boldsymbol{\upbeta}}_{\mathbf{0}} + {\left({{\boldsymbol{\upbeta}}_{\mathbf{1}}}^{\mathbf{2}} + {{\boldsymbol{\upbeta}}_{\mathbf{2}}}^{\mathbf{2}}\right)}^{\mathbf{1}/\mathbf{2}}\right\} - \mathbf{exp}\ \left\{\ {\boldsymbol{\upbeta}}_{\mathbf{0}} - {\left({{\boldsymbol{\upbeta}}_{\mathbf{1}}}^{\mathbf{2}} + {{\boldsymbol{\upbeta}}_{\mathbf{2}}}^{\mathbf{2}}\right)}^{\mathbf{1}/\mathbf{2}}\right\} $$

Sensitivity analyses were conducted in the final model using the leave-one-out cross-validation approach.

In an attempt to find associations between ILI incidence and variations in climate or days of school, meteorological data from the city of Salvador were obtained from the Brazilian national meteorological database [27]. Information on the number of monthly school days was obtained from published government data [28]. During our study, the mean weekly precipitation was 34 mm/m^2^ (SD = 51 mm/m^2^, range = 0–309 mm/m^2^). The mean weekly relative humidity and temperature were 83 % (SD = 5 %, range 72–96.5 %) and 26 **°**C (SD = 1.6 **°**C**,** range = 22–29 **°**C), respectively. On average, the month with highest precipitation and relative humidity was May, the warmest month was January, and the coolest month was July. There was a mean of 17 school days per month (SD = 6) with major school breaks occurring in January and June, coinciding with vacation periods in the Northeast of Brazil, which typically occur in June and from the middle of December to the middle of February. Regression models that controlled for seasonal confounders (annual and biannual sine and cosine variables) were used to test for associations between ILI incidence and weekly and monthly climactic variables (precipitation, median temperature, median relative humidity), and total monthly school days (Additional file 1: Figure S1).

## Results

### Characteristics of subjects

**Fig. 1:**
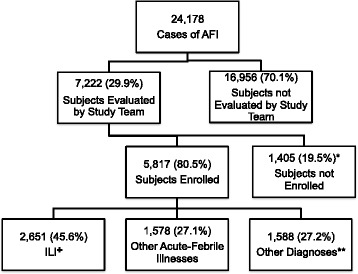
Enrollment of acute febrile illness (AFI) patients and influenza-like illness (ILI) through enhanced community-based surveillance in Pau da Lima, Salvador, Brazil, from April 1, 2009 to March 31, 2013. * Reasons for which the evaluated subjects were not enrolled included refusal (734; 52.0 %), loss of follow-up before enrollment (333; 23.7 %), minors without parents (329; 23.4 %), exclusion because of missing variables for ILI criteria (9; 0.6 %). ** Other diagnoses included dengue (1,445; 91 % [665; 46 % confirmed and 780; 54 % probable]) and leptospirosis (143; 9 % [68; 47.5 % confirmed and 75; 52.5 % probable]). ✚ 742 (27.9 %) reside within the Fiocruz cohort site

During the study period, a total of 24,178 patients presented to the SMEC with fever and 7,222 (29.9 %) were evaluated by our study team for possible enrollment. Of these, 5,817 (80.5 %) were enrolled in the AFI study (Fig. 1). The eligible subjects who were not evaluated by the study team (those seen outside of study hours) were similar to those evaluated by our study team in regard to age (median age = 20 and 18 years) and sex (47 % and 46 % male). Subjects evaluated by the study team and enrolled had a higher median age (21 years) and had a higher proportion of males (47 %) compared with eligible subjects that were evaluated by the study team and not enrolled (median age = 15 years; 44 % male *p* < 0.05).

Of the 5,817 patients enrolled, 2,651 (45.6 %) met criteria for ILI and did not have laboratory evidence for infection with dengue or leptospirosis. Of the 1,445 probable or confirmed dengue cases, 729 (50.4 %) of them met criteria for ILI (fever, cough and/or sore throat for ≤7 days) but were excluded from the ILI analysis. Of the 143 probable or confirmed leptospirosis cases, 81 (56.6 %) met the criteria for ILI but also were excluded from the ILI analyses. Characteristics of subjects with ILI are shown in Table 1. Fewer cases of ILI were admitted to the hospital (1.2 %) compared with dengue (2.3 %) or leptospirosis (7.0 %).

**Table 1:** Characteristics of patients with influenza-like illness

Characteristics	Number (%) or median (interquartile range)	
	Total cases from Pau da Lima community *N* = 2,651	Subgroup of cases from fiocruz cohort site *N* = 742
Sociodemographics		
Age in years	20 (10–30)	19 (9–30)
Male sex	1,231 (46.4)	321 (43.3)
Ethnicity		
White	220 (8.3)	53 (7.4)
Black	1,207 (45.5)	367 (51.3)
Mixed/Multiracial	1,078 (40.6)	287 (40.1)
Other	48 (1.8)	8 (1.1)
Clinical Features		
Duration of illness in days	3 (2–4)	3 (1–3)
Measured fever >37.8	1,267 (47.8)	357 (48.1)
Initial symptoms		
Cough	1,607 (60.6)	450 (60.6)
Sore throat	2,058 (77.6)	580 (78.1)
Cough and sore throat	1,014 (38.2)	288 (38.8)
Headache	2,206 (83.2)	617 (83.1)
Lethargy	2,169 (81.8)	600 (80.8)
Myalgia	1,878 (70.8)	532 (71.7)
Emesis	672 (25.3)	181 (24.4)
Diarrhea	336 (12.7)	92 (12.4)
Hospitalization	32 (1.2)	8 (1.1)
Death	3 (0.1)	1 (0.001)

### Incidence of ILI

The estimated overall mean annual incidence of ILI in the Fiocruz cohort site was 60 cases per 1,000 population/year (95 % CI = 58–62). The estimated incidences by age and by study year are shown in Table 2. The age-specific incidence was highest among children 5 to 9 years of age, with a trend of decreasing incidence with increasing age.

**Table 2:** Estimated annual incidence of influenza-like illness by age and by year

Age	Year 1	Year 2	Year 3	Year 4	Total
	Incidence per 1,000 population (95 % confidence interval)				
5–9 years	105 (92–118)	92 (80–104)	76 (65–87)	86 (75–98)	92 (86–98)
10–19 years	81 (70–92)	78 (67–89)	85 (73–96)	75 (64–85)	80 (75–85)
20–29 years	64 (55–73)	52 (44–61)	52 (43–60)	65 (56–75)	59 (55–64)
30–39 years	48 (39–58)	42 (33–51)	60 (50–71)	44 (35–53)	49 (44–54)
40–49 years	32 (23–41)	19 (12–26)	26 (18–34)	33 (24–41)	28 (24–33)
>50 years	12 (6–17)	33 (24–43)	14 (8–20)	30 (21–38)	21 (17–24)
All age groups	63 (59–67)	57 (53–61)	56 (52–60)	59 (55–64)	60 (58–62)

NOTE. Estimated annual incidence of ILI was adjusted by multiplying the age-specific total AFI cases divided by age-specific enrolled AFI cases within each surveillance year in the Fiocruz cohort Area. Study years were defined as starting on April 1^st^ and ending March 31^st^ from 4/1/2009 to 3/31/2013

### Seasonality of ILI

**Fig. 2:**
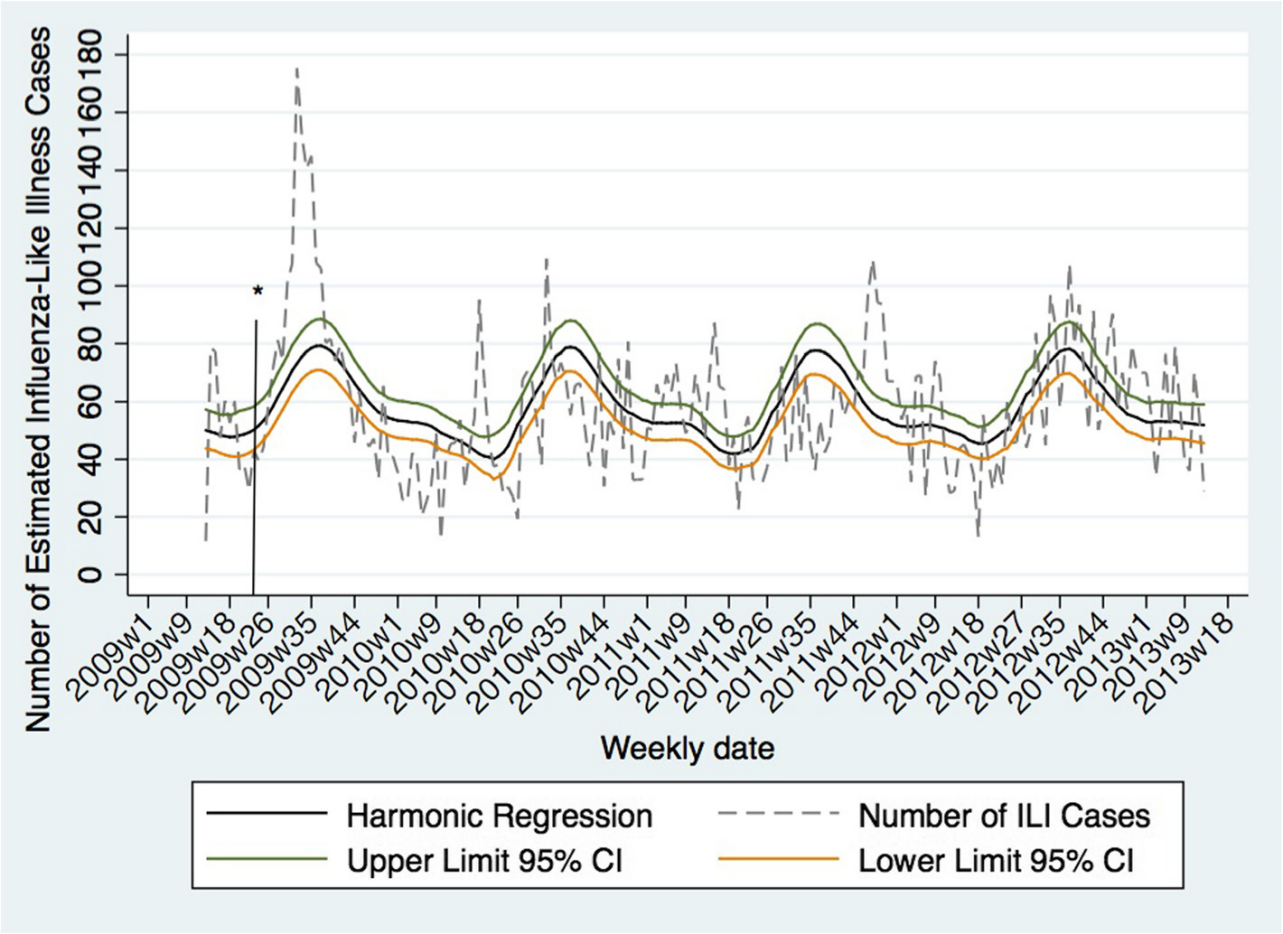
Seasonality of influenza-like illness in Pau da Lima, Salvador, Brazil, from April 1, 2009 to March 31, 2013. * First reported case of influenza A/H1N1 in Bahia, Brazil in 2009

**Fig. 3:**
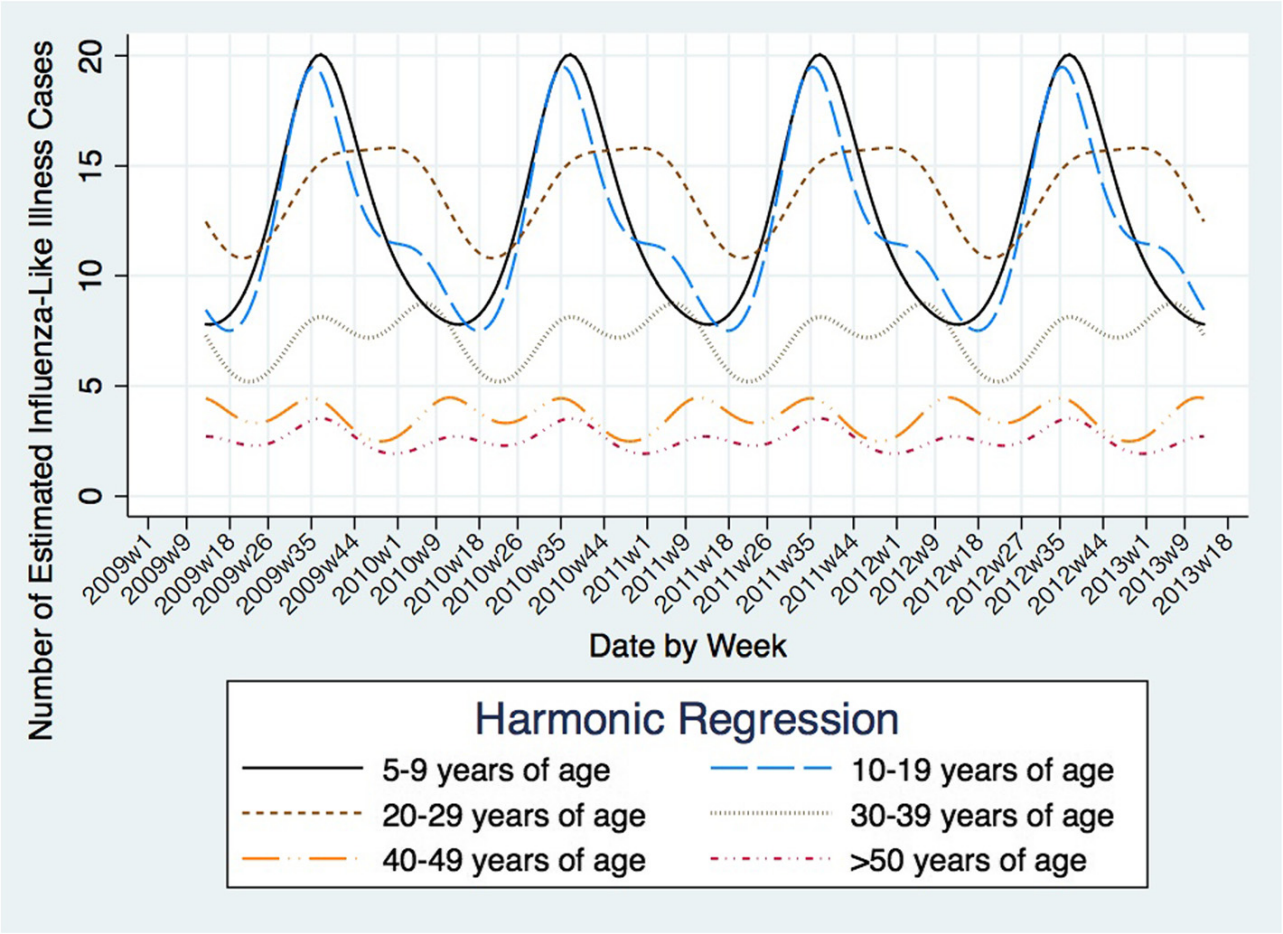
Seasonality of influenza-like illness by age group in Pau da Lima, Salvador, Brazil, from April 1, 2009 to March 31, 2013

The average epidemic peak had an estimated 74 cases per week. More than double the number of cases occurred during peak weeks compared to nadir weeks. The observed seasonal pattern of ILI was different than the dengue seasonal pattern. Therefore, it is likely that the observed seasonality of ILI was not strongly influenced by occasional dengue cases that the laboratory investigation may have missed. ILI peaks consistently occurred annually between August and September (weeks 35 to 40) (Fig. 2), while dengue epidemic peaks occurred between May and June (weeks 20 to 25). The seasonal pattern of ILI subdivided by age groups demonstrated that on average, the annual peak intensity (from peak to nadir) was higher in children 5–9 years of age and occurred five weeks earlier in 5–9 year-olds than in 20–29 year-olds (Fig. 3). Sensitivity analyses were conducted in the final model using the leave-one-season-out cross-validation approach. This analysis did not reveal a significant qualitative difference in the epidemic curves with the exception of the first study year, which coincided with the 2009 H1N1 influenza pandemic. When the first study year was removed, the overall seasonal curve became less intense and shifted forward. Removing any other year from the model did not significantly alter the epidemic curves (Additional file 1: Figure S2).

Time series depicting the temporal associations between ILI and dengue, monthly precipitation, monthly relative humidity, monthly temperature and monthly days of school are shown in Additional file 1: figures S3-S7. After using a negative binomial regression and adjusting for the known confounders (annual and biannual sine and cosine variables), no statistically significant association was seen between the incidence of ILI and weekly precipitation (RR = 1.00; 95 % CI = 0.99–1.00; *p* = 0.59), median weekly temperature (RR = 0.98; 95 % CI = 0.93–1.05; *p* = 0.70), or weekly relative humidity (RR = 1.00; 95 % CI = 0.99–1.02; *p* = 0.75). We detected a statistically significant positive association between monthly days of school and ILI incidence in children 5–9 years of age, even after adjusting for the sine and cosine variables (RR = 1.04; 95 % CI = 1.00 to 1.09; *p* = 0.04). Every additional day of school in the month corresponded with a 4 % increase (95 % CI = 0–9 %) in the incidence of ILI in children 5–9 years of age. When this analysis was repeated with older children and adults, no statistically significant associations were found.

## Discussion

Using data collected during a four-year study, we identified a significant ILI burden in a tropical urban community of Brazil. The estimated incidence of ILI in our study was substantial, with a mean of 60 cases per 1,000 persons per year in all age groups. These findings are similar to previously published data about the burden of ILI in other countries, such as subtropical southern China, with rates of ILI of 72 per 1,000 persons [29] and Kenya, a tropical country, with 64 cases per 1,000 person-years [30]. The incidence of ILI in our study was particularly high in children and adolescents from 5–9 years of age with rates as high as 105 cases per 1,000 persons per year during the 2009 H1N1 season. The incidence of ILI among children in our study was only slightly lower than rates of ILI in children residing in temperate climates, where reported incidence is as high as 118 per 1,000 persons per year [31]. These data suggest that ILI is a major problem in the tropics, particularly in children.

On April 25, 2009, the first case of a novel influenza A/H1N1 was reported in Brazil. It is estimated that there were over 2,000 deaths in Brazil during the epidemic that followed, and the country endured one of the highest mortality rates from swine flu in the world [32]. Additionally, it was observed that children and adolescents had the highest risk of infection during this outbreak [2]. Our data were consistent with these findings, with the highest intensity of the ILI peaks occurring during the 2009 cycle and with children disproportionally affected when compared with the elderly. In our study the peak incidence of ILI was earlier and more dramatic in children among whom peak incidence occurred several weeks prior to the peaks in older patients. These findings are similar to previously published data in temperate climates and suggest that infection in children may be important in the propagation of annual epidemics in the tropics as well as in temperate climates [26].

Multiple respiratory pathogens have been associated with ILI. However, little is known regarding the seasonality of these respiratory pathogens, particularly in the tropics. Influenza and RSV have been the only two ILI-associated pathogens that have been found to have a consistent seasonal component in the tropics of Brazil [13, 19]. Our study identified a clear and distinct seasonal pattern of syndromic ILI in this urban community of Salvador, Brazil. Many methods have been used to assess seasonality of infectious diseases [33]. Our approach was based on the harmonic regression model, a widely accepted model that fits a sine and cosine curve to a time series of incident cases using regression. Using a regression model with harmonic terms, we found that ILI had an annual cyclical component with peaks usually occurring between August and September.

The seasonal variation of our study differs from previously published reports from Brazil. Freitas (2013) found no influenza seasonality in the northeastern tropical portion of the country and only found seasonal peaks of RSV infection between April and June [34]. The difference in our findings might be due to sampling variation, patient selection, or the sociodemographics of the study population. The Freitas study used data collected from sentinel surveillance units in Brazil where five weekly samples were sent for analysis for respiratory viruses, yet no standardized protocol was used to select patients and only 1 % of all persons with ILI had samples collected.

Two other studies [35, 36] evaluated children with respiratory illness in Fortaleza, a northern tropical city in Brazil located 1,000 kilometers north of Salvador. These studies demonstrated marked seasonality of influenza, yet they found wide yearly variation in epidemic months. Peaks occurred between February and June, several months prior to the peaks in our study. In 2007, Alonso et al., first described the different timing of influenza epidemics in Brazil [9]. His study evaluated influenza mortality from 1997 to 2001 and found that mortality had a seasonal southward wave, originating in equatorial states in April and moving toward the southern part of the country during June and July. The differences in the timing of the epidemic peaks between our study and previously published influenza literature have various plausible explanations. First, our study did not assess laboratory-confirmed influenza or influenza mortality, but rather ILI, which could include other respiratory viral infections. Furthermore, our study included data after the 2009 influenza A/H1N1 pandemic, which could have shifted the epidemic curve later in the year. However, our findings are consistent with a recent study, which evaluated the frequency of respiratory samples positive for pandemic influenza in a pediatric hospital in Salvador, Brazil in 2009 [37]. Although not a population-based investigation, this study similarly found that the highest frequency of influenza occurred between August and September. The marked differences in the timing of influenza epidemics through different parts of Brazil throughout the last decade highlight the importance of annual surveillance, since yearly variations in seasonality can have significant ramifications when considering appropriate timing for influenza immunization campaigns.

Several other studies have attempted to link incidence of influenza in the tropics with meteorological factors. Strong associations with high rainfall and relative humidity have been noted in Singapore [11], Senegal [14], Bangladesh [38], Cambodia [39], and northern Brazil [13]. In contrast, our data indicate that the peaks in ILI were not associated with high precipitation, relative humidity, or low temperature. Our approach had several strengths. We used a regression model that adjusted for the seasonal variables. We used this method because the seasonal nature of the variables themselves can be confounders. If the exposure has a seasonal component (such as rain, temperature, relative humidity, or days in school) and the outcome also has seasonality, then the seasonal nature of the variables can bias the results. Our findings may have differed from previously published literature given that we controlled for this aspect of seasonality. In addition, we used a syndromic case definition, which reflects the incidence of multiple possible respiratory pathogens and not only influenza; hence, the lack of association between the incidence of ILI and meteorological factors may be due to the use of a syndromic case definition rather than virologically confirmed influenza.

Several studies in temperate climates have demonstrated that on average, school closures are associated with decreases in ILI (16–40 % reduction) [40–42], though none have demonstrated this in tropical climates. Our study found a significant association between the incidence of ILI in children 5–9 years of age and the total days of school per month. This suggests that the close and frequent contact of children in school is likely playing a role in propagating epidemics of ILI in the tropics.

Our analyses have several limitations. First, we likely underestimated the rates of ILI cases given that approximately 15 % of patients in the community under surveillance may seek medical attention at a different facility. Additionally, children and adolescents 5–19 years of age were underrepresented within the enrolled group compared with subjects not enrolled. Nevertheless, our rates were age-adjusted for the proportion that was not sampled in order to minimize this underestimation. The age adjustments do assume that the incidence of ILI outside of study hours approximates the incidence within study hours; however, this assumption may not hold true in the real world. Second, we used a clinical case definition for ILI and did not test respiratory samples for viruses. Even though ILI is a well-established marker for viral respiratory infections, the estimated specificity for influenza can vary year to year. Given this, our data reflect the incidence and seasonal patterns of multiple respiratory pathogens, including but not exclusively influenza. One of the strengths of our study is that we ruled out dengue and leptospirosis in our cases, which likely increased the specificity of our case definition in this population. On the other hand, co-infections with dengue or leptospirosis and a respiratory virus would have incorrectly excluded them as ILI cases, potentially underestimating the true incidence of ILI in this community. However, coinfection of ILI and dengue or leptospirosis is improbable given their different seasonality patterns and different routes of infection, making unlikely that exclusion of dengue or leptospirosis cases had a substantial impact in our ILI incidence estimates. Third, we enrolled patients for only 42 h per week; therefore, we did not capture all episodes of ILI in the community. We did adjust for the times during which we did not sample; however, as mentioned before, our adjustment assumes that the rates of ILI are similar during the weekdays, weeknights, and weekends. Lastly, our study analyzed data for only four years, which is a relatively short period to assess seasonality.

## Conclusions

The main finding of this four-year community-based study is that there is a significant burden and clear seasonality of ILI in a poor urban community of Salvador, Brazil, particularly among children and adolescents 5–19 years of age. This suggests that ILI is a public health threat not only in temperate countries, but in tropical countries as well. Seasonal peaks of ILI in children 5–9 years of age are associated with the number of school days per month. Understanding the burden, seasonality and potential drivers of ILI is important when considering timing for influenza vaccination campaigns in Brazil. Further research, particularly using laboratory-confirmed influenza and its seasonal drivers is needed in the tropics of Brazil.

## Additional file

Additional file 1: Supplementary Study Data. (DOCX 483 kb)

## Abbreviations

ILI: influenza-like illness
CDC: centers for disease control and prevention
WHO: world health organization
AFI: acute febrile illness
SMEC: são marcos emergency center
RT-PCR: reverse transcriptase-polymerase chain reaction
MAT: microscopic agglutination tests
ELISA: enzyme-linked immunoabsorbent assay
IgM: immunoglobulin M
NS1: non-structural protein 1
95 % CI: 95 % confidence intervals
SD: standard deviation

## References

[1] World Health Organization. Influenza (Seasonal) Fact Sheet N°211. 2013. http://www.who.int/mediacentre/factsheets/fs211/en/. Accessed 15 Dec 2014.

[2] Van Kerkhove MD, Vandemaele KA, Shinde V, Jaramillo-Gutierrez G, Koukounari A, Donnelly CA (2011). Risk factors for severe outcomes following 2009 influenza A (H1N1) infection: a global pooled analysis. PLoS Med.

[3] Nair H, Brooks WA, Katz M, Roca A, Berkley JA, Madhi SA (2011). Global burden of respiratory infections due to seasonal influenza in young children: a systematic review and meta-analysis. Lancet.

[4] Centers for Disease C, Prevention (2013). Influenza activity--United States, 2012–13 season and composition of the 2013–14 influenza vaccine. MMWR Morb Mortal Wkly Rep.

[5] Hirve S, Chadha M, Lele P, Lafond KE, Deoshatwar A, Sambhudas S (2012). Performance of case definitions used for influenza surveillance among hospitalized patients in a rural area of India. Bull World Health Organ.

[6] Gupta V, Dawood FS, Rai SK, Broor S, Wigh R, Mishra AC (2013). Validity of clinical case definitions for influenza surveillance among hospitalized patients: results from a rural community in North India. Influenza Other Respi Viruses.

[7] Murray EL, Khagayi S, Ope M, Bigogo G, Ochola R, Muthoka P (2013). What are the most sensitive and specific sign and symptom combinations for influenza in patients hospitalized with acute respiratory illness? Results from western Kenya, January 2007-July 2010. Epidemiol Infect.

[8] Bloom-Feshbach K, Alonso WJ, Charu V, Tamerius J, Simonsen L, Miller MA (2013). Latitudinal variations in seasonal activity of influenza and respiratory syncytial virus (RSV): a global comparative review. PLoS One.

[9] Alonso WJ, Viboud C, Simonsen L, Hirano EW, Daufenbach LZ, Miller MA (2007). Seasonality of influenza in Brazil: a traveling wave from the Amazon to the subtropics. Am J Epidemiol.

[10] Surveillance WPRGI, System R (2012). Epidemiological and virological characteristics of influenza in the Western Pacific Region of the World Health Organization, 2006–2010. PLoS One.

[11] Lee VJ, Yap J, Ong JB, Chan KP, Lin RT, Chan SP (2009). Influenza excess mortality from 1950–2000 in tropical Singapore. PLoS One.

[12] Tamerius JD, Shaman J, Alonso WJ, Bloom-Feshbach K, Uejio CK, Comrie A (2013). Environmental predictors of seasonal influenza epidemics across temperate and tropical climates. PLoS Pathog.

[13] de Arruda E, Hayden FG, McAuliffe JF, de Sousa MA, Mota SB, McAuliffe MI (1991). Acute respiratory viral infections in ambulatory children of urban northeast Brazil. J Infect Dis.

[14] Dosseh A, Ndiaye K, Spiegel A, Sagna M, Mathiot C (2000). Epidemiological and virological influenza survey in Dakar, Senegal: 1996–1998. Am J Trop Med Hyg.

[15] Chew FT, Doraisingham S, Ling AE, Kumarasinghe G, Lee BW (1998). Seasonal trends of viral respiratory tract infections in the tropics. Epidemiol Infect.

[16] Lowen AC, Steel J (2014). Roles of humidity and temperature in shaping influenza seasonality. J Virol.

[17] Garza RC, Basurto-Davila R, Ortega-Sanchez IR, Carlino LO, Meltzer MI, Albalak R (2013). Effect of winter school breaks on influenza-like illness, Argentina, 2005–2008. Emerg Infect Dis.

[18] Campos EC, Sudan LC, Mattos ED, Fidelis R (2012). Factors associated with influenza vaccination among the elderly: a cross-sectional study in Cambe, Parana State, Brazil. Cad Saude Publica.

[19] Straliotto SM, Siqueira MM, Muller RL, Fischer GB, Cunha ML, Nestor SM (2002). Viral etiology of acute respiratory infections among children in Porto Alegre, RS, Brazil. Rev Soc Bras Med Trop.

[20] The Brazilian Institute of Geography and Statistics. IBGE-cities. 2010. www.ibge.gov.br/cidadesat/default.php. Accessed 11 Sept 2014.

[21] Kikuti M, Cunha GM, Paploski IA, Kasper AM, Silva MM, Tavares AS (2015). Spatial distribution of dengue in a Brazilian urban slum setting: Role of socioeconomic gradient in disease risk. PLoS Negl Trop Dis.

[22] Reis RB, Ribeiro GS, Felzemburgh RD, Santana FS, Mohr S, Melendez AX (2008). Impact of environment and social gradient on Leptospira infection in urban slums. PLoS Negl Trop Dis.

[23] Shei A, Costa F, Reis MG, Ko AI (2014). The impact of Brazil's Bolsa Familia conditional cash transfer program on children's health care utilization and health outcomes. BMC Int Health Hum Rights.

[24] Lanciotti RS, Calisher CH, Gubler DJ, Chang GJ, Vorndam AV (1992). Rapid detection and typing of dengue viruses from clinical samples by using reverse transcriptase-polymerase chain reaction. J Clin Microbiol.

[25] Merrill RM. Introduction to epidemiology. 6th ed. Burlington, Mass. Sudbury, MA: Jones & Bartlett Learning; 2013.

[26] Lofgren ET, Wenger JB, Fefferman NH, Bina D, Gradus S, Bhattacharyya S (2010). Disproportional effects in populations of concern for pandemic influenza: insights from seasonal epidemics in Wisconsin, 1967–2004. Influenza Other Respi Viruses.

[27] Instituto Nacional de Meterologia. Salvador, BA, Brasil: INMET. http://www.inmet.gov.br/portal/. Accessed 11 Sept 2014.

[28] Secretaria da Educação Calendário de Estudante-Dias Letivos. Salvador, BA, Brazil. http://www.educacao.salvador.ba.gov.br. Accessed 11 Sept 2014.

[29] Guo RN, Zheng HZ, Li JS, Sun LM, Li LH, Lin JY (2011). A population-based study on incidence and economic burden of influenza-like illness in south China, 2007. Public Health.

[30] Katz MA, Lebo E, Emukule G, Njuguna HN, Aura B, Cosmas L (2012). Epidemiology, seasonality, and burden of influenza and influenza-like illness in urban and rural Kenya, 2007–2010. J Infect Dis.

[31] Fowlkes A, Giorgi A, Erdman D, Temte J, Goodin K, Di Lonardo S (2014). Viruses associated with acute respiratory infections and influenza-like illness among outpatients from the Influenza Incidence Surveillance Project, 2010–2011. J Infect Dis.

[32] Yokota RT, Skalinski LM, Igansi CN, de Souza LR, Iser BP, Reis PO (2011). Risk factors for death from pandemic (H1N1) 2009, southern Brazil. Emerg Infect Dis.

[33] Christiansen CF, Pedersen L, Sorensen HT, Rothman KJ (2012). Methods to assess seasonal effects in epidemiological studies of infectious diseases--exemplified by application to the occurrence of meningococcal disease. Clin Microbiol Infect.

[34] Freitas FT (2013). Sentinel surveillance of influenza and other respiratory viruses, Brazil, 2000–2010. Braz J Infect Dis.

[35] Alonso WJ, Laranjeira BJ, Pereira SA, Florencio CM, Moreno EC, Miller MA (2012). Comparative dynamics, morbidity and mortality burden of pediatric viral respiratory infections in an equatorial city. Pediatr Infect Dis J.

[36] Moura FE, Perdigão AC, Siqueira MM (2009). Seasonality of influenza in the tropics: a distinct pattern in northeastern Brazil. Am J Trop Med Hyg.

[37] Silva RC, Siqueira MA, Netto EM, Bastos JS, Nascimento-Carvalho CM, Vilas-Boas AL (2014). Epidemiological aspects of influenza A related to climatic conditions during and after a pandemic period in the city of Salvador, Northeastern Brazil. Mem Inst Oswaldo Cruz.

[38] Imai C, Brooks WA, Chung Y, Goswami D, Anjali BA, Dewan A (2014). Tropical influenza and weather variability among children in an urban low-income population in Bangladesh. Glob Health Action.

[39] Mardy S, Ly S, Heng S, Vong S, Huch C, Nora C (2009). Influenza activity in Cambodia during 2006–2008. BMC Infect Dis.

[40] Chowell G, Towers S, Viboud C, Fuentes R, Sotomayor V (2014). Rates of influenza-like illness and winter school breaks, Chile, 2004–2010. Emerg Infect Dis.

[41] Cauchemez S, Ferguson NM, Wachtel C, Tegnell A, Saour G, Duncan B (2009). Closure of schools during an influenza pandemic. Lancet Infect Dis.

[42] Chao DL, Halloran ME, Longini IM (2010). School opening dates predict pandemic influenza A(H1N1) outbreaks in the United States. J Infect Dis.

