# Supplementary material for: Influenza-like illness in an urban community of Salvador, Brazil: incidence, seasonality and risk factors
Source: BMC Infect Dis. 2016 Mar 15;16:125. doi: 10.1186/s12879-016-1456-8 (PMC4791800; doi:10.1186/s12879-016-1456-8)
Supplement: Additional file 1: — Supplementary Study Data. (DOCX 483 kb) [file 12879_2016_1456_MOESM1_ESM.docx]

**Supplementary Data**

| **Table S1.** Candidate models. “X” indicates that the covariate was included. The AIC scores are given for each model. Models 1-8 represent weekly incidences whereas Models 9-16 represent monthly incidences. The model with the lowest AIC score is shaded gray | | | | | |
| --- | --- | --- | --- | --- | --- |
| ***Model*** | ***Annual harmonic*** | ***Semi-annual harmonic*** | ***Dengue*** | ***ILI*** | ***AIC Score*** |
| ***1*** | **X** | ***X*** | **X** | ***X*** | 2653.76 |
| ***2*** |  | **X** | **X** | **X** | 2881.61 |
| ***3*** | **X** |  | **X** | **X** | 2696.42 |
| ***4*** | **X** | **X** |  | **X** | 2687.17 |
| ***5*** |  |  | **X** | **X** | 2913.60 |
| ***6*** |  |  |  | **X** | 3057.65 |
| ***7*** |  | **X** |  | **X** | 2988.92 |
| ***8*** | **X** |  |  | **X** | 2751.12 |
| ***9*** | **X** | ***X*** | **X** | ***X*** | 1261.51 |
| ***10*** |  | **X** | **X** | **X** | 1523.65 |
| ***11*** | **X** |  | **X** | **X** | 1323.82 |
| ***12*** | **X** | **X** |  | **X** | 1276.50 |
| ***13*** |  |  | **X** | **X** | 1587.12 |
| ***14*** |  |  |  | **X** | 1699.01 |
| ***15*** |  | **X** |  | **X** | 1602.28 |
| ***16*** | **X** |  |  | **X** | 1360.58 |

**Figure S1.**

**Incidence = exp{β_0_+ β_1__cos(Ø)+ β_2__sin(Ø) + β_3__cos(Φ)+β_4__sin(Φ) + β_5__AssociatedV + ε}**

Where β_0_ represents the intercept or baseline level of ILI; β_1_ – β_4_ are the coefficients of the harmonic; Ø =2π*month/12 Φ=2π*month/6 (representing annual and biannual monthly epidemics) and β_5_ represents the variable being tested for association with the ILI incidence and included: monthly precipitation, monthly median temperature, monthly median relative humidity, and total monthly school days.

Relative risks were estimating by taking the exponent of the coefficient β5. The corresponding confidence intervals for relative risks were similarly transformed.

**Figure S2. Sensitivity analysis showing the leave-one-out cross validation**

**Figure S3. Influenza-like illness cases and monthly days of school.**

**Figure S4. Influenza-like illness cases and total monthly precipitation.**

**Figure S5. Influenza-like illness cases and monthly median relative humidity.**

**Figure S6. Influenza-like illness cases and monthly median temperature.**

**Figure S7. Influenza-like illness and dengue cases.**

|  |
| --- |
